# World Cancer Day 2014: "Increasing The Awareness"

**Published:** 2014-10-04

**Authors:** Hamid Nasri, Mahmoud Rafieian-Kopaei

**Affiliations:** 1 Department of Nephrology, Division of Nephropathology, Isfahan University of Medical Sciences, Isfahan, Iran; 2 Medical Plants Research Center, Shahrekord University of Medical Sciences, Shahrekord, Iran

**Keywords:** World Health Organization, Cancer, Patients

World Cancer Day (WCD) is celebrated on February 4^th^ each year around the world to remind the efforts done by united nations, World Health Organization (WHO), and other governmental and non-governmental health organizations with the aim of delivering the real message about cancer and its treatments to fight against this fatal disease through uniting all the people on global basis (1-4). In brief, cancer is a large group of different disorders involving unregulated cell growth. In malignancy, cells divide and grow uncontrollably to form malignant tumors and to invade neighboring parts of the body. The cancer tumor may spread to more distant parts of the body through blood or lymphatic systems (5-8). It has been observed that most of the cancer cases and related deaths happen in less developed countries that this situation is expected to get worse by 2030. Therefore, it is very crucial to get control over such condition throughout the world. Furthermore, WCD aims to save millions of preventable deaths every year by raising alertness and by educating people about malignancy, while forcing the governments throughout the world to take action against this disease (2-8). The day is also a key chance for cancer patients to ensure that world leaders stick to the promises they made at the United Nations Summit for reducing the cancer and its impacts. During this particular occasion, participants try to promote healthy lifestyles, balanced diet, regular physical activity, weight management, as well as using antioxidants in order to diminish the risk of malignancies (1-8). Furthermore, this day is celebrated to plan certain new strategies and to imply various new programs in order to make people aware of this disorder. WCD is also celebrated to make non-patient people aware about the preventive methods and the risk factors of cancer (2-8). The theme of WCD of 2014 is "Debunk the Myths" because some people believe that if they contact or live with a patient who has cancer, they would get cancer as well. The day is, therefore, distinguished to eradicate such types of the social myths (5-13) and to make certain concepts about different aspects of the malignancy, such as its symptoms, causing factors and treatment (1, 3, 9). Furthermore, on this day, some of activities are organized to indicate that cancer patients should not be treated distinctly, while they are entitled to the same rights as normal people in the society (14-19). Although they have less chances of existence, they should be fulfilled their wishes by their relatives. It should not make them sense that the treatment remedies are given them for existence as they are dying. In this regards, it is very important to make them feel better like a normal person. They should be also prepared a normal environment in their home and society. It is essential that normal people avoid being overly sympathetic to them (20-27).
